# Numerical evaluation of cutting strategies for thin-walled parts

**DOI:** 10.1038/s41598-024-51883-1

**Published:** 2024-01-17

**Authors:** Andreas Andersson Lassila, Daniel Svensson, Wei Wang, Tobias Andersson

**Affiliations:** School of Engineering Science, University of Skövde, Kaplansgatan 11, SE-541 34 Skövde, Sweden

**Keywords:** Mechanical engineering, Engineering

## Abstract

Static form errors due to in-process deflections is a major concern in flank milling of thin-walled parts. To increase both productivity and part geometric accuracy, there is a need to predict and control these form errors. In this work, a modelling framework for prediction of the cutting force-induced form errors, or thickness errors, during flank milling of a thin-walled workpiece is proposed. The modelled workpiece geometry is continuously updated to account for material removal and the reduced stiffness matrix is calculated for nodes in the engagement zone. The proposed modelling framework is able to predict the resulting thickness errors for a thin-walled plate which is cut on both sides. Several cutting strategies and cut patterns using constant z-level finishing are studied. The modelling framework is used to investigate the effect of different cut patterns, machining allowance, cutting tools and cutting parameters on the resulting thickness errors. The framework is experimentally validated for various cutting sequences and cutting parameters. The predicted thickness errors closely correspond to the experimental results. It is shown from numerical evaluations that the selection of an appropriate cut pattern is crucial in order to reduce the thickness error. Furthermore, it is shown that an increased machining allowance gives a decreased thickness error for thin-walled plates.

## Introduction

Thin-walled components characterized by a large ratio of height to thickness and tight tolerances are typical lightweight designs adopted by the aerospace industry. In the current shift to electro-mobility (E-mobility), they are also widely applied in part design to achieve a balance between structural strength and weight, e.g. the stator housing and the housing of battery packs. Although alternative manufacturing techniques such as additive manufacturing have attracted more attention, peripheral milling/flank milling remains dominant for thin-walled components machining.

Machining of thin-walled components is still challenging due to the difficulties (D1–D3) summarized as follows. (D1) Owing to the low stiffness, the deflections of the cutting tool and the workpiece caused by cutting forces results in undercuts as the actual width of cut is smaller than the nominal value. (D2) During material removal, the stiffness of the workpiece becomes even lower which aggravate the in-process deflections. (D3) The forced vibrations and chatter cause poor surface quality and even cutting tool breakages. D1 and D2 are cutting force induced static form errors that affect the machining accuracy as they leave surface location errors (SLE) while D3 is vibration induced errors, i.e. dynamic errors.

To address these issues, many approaches have been proposed. (1) Selection of appropriate cutting tools. In order to avoid the interference between the upper portion of wall and the tool shank, a tool with relieved shank was applied^1^. The helical angle of cutting edges has a significant influence on the cutting stability when using a small width of cut in combination with a low feed rate^2^. The size of the corner radius affects the cutting force distribution between axial and radial directions and selecting an appropriate corner radius is important to limit the in-process deflections^3^. In order to suppress vibrations, cutting tools equipped with mass dampers can be used^4^. (2) Selection of appropriate cutting parameters. As the feed rate strongly affects the cutting forces, feed rate scheduling is a common practical method to control the instantaneous cutting force and thereby also the in-process deflections^5^. Varying the depth of cut is another effective strategy and in^6^, a variable depth-of-cut multi-axis machining strategy based on workpiece deflection constraints was developed. Similarly, the radial width of cut in finishing operation is also controlled to limit the in-process deflections^7^. When the machining allowance left by the roughing operation is removed by a single pass the machining allowance is equal to the width of cut. A large machining allowance gives a higher local stiffness of the workpiece at the cutting point but an increased cutting force, therefore a compromised machining allowance should be selected^3,8^. Moreover, the spindle speed should be carefully tuned to avoid tooth-passing frequencies that are close to the workpiece natural frequencies^9^. (3) Choose optimal cut pattern. To reduce the non-cutting time, the side-by-side cut pattern is normally applied to remove the materials on both sides of the workpiece. Regarding thin-walled structures, a higher local stiffness can be achieved by applying the waterline or the jump-to-jump cut pattern^3^. (4) Tool path compensation. As deflections of the cutting tool and the workpiece cause over/undercut, the toolpath points should be adjusted accordingly so that the desired width of cut is obtained^10^. (5) Optimization of the cutting sequence. Apart from cut pattern, the material removal sequence can also be planned to improve the local stiffness during cutting. For instance, in^11^, the material removal volumes are divided into small blocks which are gradually added to the final part model to generate the in-process workpiece. When the cutting force is applied, the in-process deflections can be controlled to be smaller than a given threshold value by adding blocks of material. As a result, the cutting sequencing is obtained by reversing the adding sequencing of blocks. (6) Design of special fixtures. In order to provide extra support, intelligent fixtures are designed to enhance the local stiffness during cutting. For example, wax^12^ or ice^13^ are filled in the deep and narrow space to provide extra support as they could easily be removed by the cutting tool. Recent overviews related to fixtures can be found in^14,15^. Additionally, high-speed machining (HSM) is popularly applied for the machining of aircraft structural components characterized by deep and thin-walled structures. The interpolation type of toolpath generated by CAM software should be tuned with numerical control unit (NCU) and axis performance of machines to achieve surface quality requirement^16^.

As demonstrated above, substantial work has been done to overcome the challenges of thin-walled part machining. The solution space has been explored in either an individual way or in a mixed manner. However, process planners are still struggling to choose appropriate cutting tools, cutting parameters, fixturing solutions and cut patterns to achieve a balance between machining quality and manufacturing cost. The trial-and-error methodology remains the common approach in the industry. Along with the development of computational methods and high-performance PC:s, modelling and simulation have attracted significant attention from both the research community and the industry. Benefiting from simulations, the machining quality in the form of SLE:s of thin-walled structures under different machining conditions can be investigated with limited physical experiments. As a result, both time and cost can be saved. Simulations of thin-walled part machining are usually realized using a hybrid approach. The cutting forces are calculated either analytically or using a meta model^17^. Finite element models of the thin-walled part are created that reflect the material removal process and its continuous effect on the workpiece stiffness. By applying the cutting force to the finite element model of the in-process workpiece and the cutting tool model, the deflections of the cutting tool and the workpiece can be computed in an iterative manner. Finally, the deflections of the cutting tool and the workpiece are used to predict the generated SLE.

The reported work has provided valuable insights on simulation of thin-walled component machining. A detailed literature overview is given in Table 1, in which “$\times$” is used to mark that the corresponding variable in “Process Plan” column is fixed in simulations, and “$\checkmark$” presents that the corresponding variable varies in simulations. In “Cutting Process” column, “$\checkmark$” is used to mark that the deflections of cutting tool/workpiece, and the effect of material removal process on stiffness reduction is considered respectively. If the answer is no, “$\times$” is assigned accordingly. Although the effects of the cutting tool and cutting parameters have been studied, studies on the effect of different cut patterns are sparsely reported. In fact, selection of an optimized cut pattern can strongly improve the machining efficiency and quality^3,18,19^. Therefore, a modelling framework is developed in this work which is capable of varying the cutting tool, fixturing solution, cutting parameters and cut pattern so that the machining process of a thin-walled part can be planned in a comprehensive way. As 40–70% of the machining errors are cutting force-induced errors^20^, this work focuses on static form error prediction.

The remaining part of this paper is structured as follows. The milling process of the considered thin wall component is described in “Milling process of a thin walled component”. The developed modelling framework and the subsequent model calibration are presented in “Modelling framework” and “Model calibration”, respectively. The experimental series used for model verification and the comparison between experimental results and simulations are given in “Experimental series” and “Comparison of experimental results with simulations”, respectively. “Numerical evaluation of cutting strategies” presents a numerical evaluation of cutting strategies, and in “Results” the results of this evaluation are given. A discussion related to the results is given in “Discussion”, and finally, the conclusions of the present study are given in “Conclusions”.

**Table 1:** Overview of simulation work for flank milling of thin-walled parts.

Ref.	Process plan						Cutting process		
	Cutter	Cutting parameters				Cut pattern	Deflections		Material removal
		$a_p$	$a_e$	$f_z$	N		Cutter	Workpiece	
^21^	$\checkmark$	$\checkmark$	$\checkmark$	$\checkmark$	$\times$	$\times$	$\checkmark$	$\checkmark$	$\checkmark$
^22^	$\times$	$\checkmark$	$\checkmark$	$\checkmark$	$\times$	$\times$	$\times$	$\checkmark$	$\checkmark$
^23^	$\times$	$\times$	$\checkmark$	$\checkmark$	$\times$	$\times$	$\times$	$\checkmark$	$\checkmark$
^24^	$\checkmark$	$\checkmark$	$\checkmark$	$\checkmark$	$\checkmark$	$\times$	$\checkmark$	$\checkmark$	$\checkmark$
^25^	$\times$	$\times$	$\checkmark$	$\checkmark$	$\checkmark$	$\times$	$\times$	$\checkmark$	$\checkmark$
^26^	$\checkmark$	$\checkmark$	$\checkmark$	$\checkmark$	$\checkmark$	$\times$	$\times$	$\checkmark$	$\checkmark$
^27^	$\times$	$\checkmark$	$\times$	$\checkmark$	$\checkmark$	$\times$	$\times$	$\checkmark$	$\checkmark$
^18^	$\times$	$\times$	$\checkmark$	$\checkmark$	$\checkmark$	$\times$	$\times$	$\checkmark$	$\checkmark$
^28^	$\checkmark$	$\checkmark$	$\times$	$\checkmark$	$\checkmark$	$\times$	$\checkmark$	$\checkmark$	$\checkmark$
^29^	$\times$	$\times$	$\times$	$\times$	$\times$	$\times$	$\times$	$\checkmark$	$\checkmark$
^30^	$\times$	$\times$	$\times$	$\times$	$\times$	$\times$	$\checkmark$	$\checkmark$	$\checkmark$
^31^	$\times$	$\times$	$\times$	$\times$	$\times$	$\times$	$\checkmark$	$\checkmark$	$\checkmark$
^32^	$\times$	$\times$	$\times$	$\times$	$\times$	$\times$	$\checkmark$	$\checkmark$	$\checkmark$
^33,34^	$\checkmark$	$\checkmark$	$\checkmark$	$\checkmark$	$\checkmark$	$\times$	$\times$	$\checkmark$	$\checkmark$
^35^	$\times$	$\checkmark$	$\checkmark$	$\times$	$\times$	$\times$	$\checkmark$	$\checkmark$	$\checkmark$
^20,36^	$\checkmark$	$\checkmark$	$\checkmark$	$\checkmark$	$\checkmark$	$\times$	$\checkmark$	$\checkmark$	$\checkmark$
^37^	$\times$	$\times$	$\times$	$\times$	$\times$	$\times$	$\times$	$\checkmark$	$\checkmark$
^38^	$\times$	$\times$	$\times$	$\times$	$\times$	$\times$	$\checkmark$	$\checkmark$	$\checkmark$

The cutting parameters $a_p$, $a_e$, $f_z$ and *N* denote axial depth of cut, radial width of cut, feed per flute and spindle speed, respectively.

## Milling process of a thin walled component

The workpieces are thin aluminum plates of 75 mm height and 120 mm length that are clamped along the bottom edge while the remaining three edges are free. The free height of the cantilevered plates is 55 mm. Since the radial width of cut is varied between experiments and the final nominal thickness is to be 3 mm for all samples, the initial thickness is varied accordingly between samples.

### Cutting strategies

**Figure 1:**
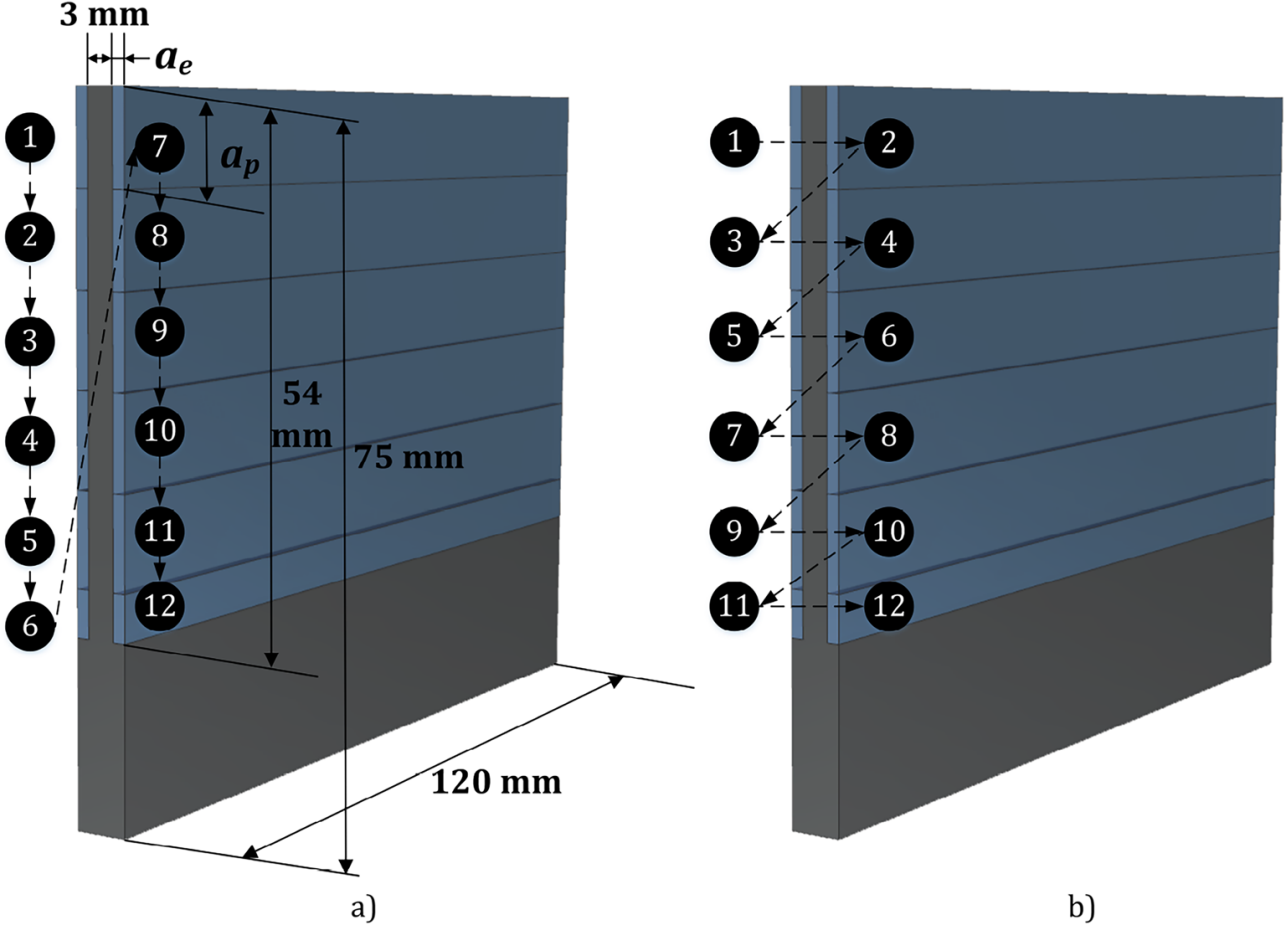
Cut patterns: SBS (**a**). WL (**b**).

Two cut patterns are considered; side-by-side (SBS) and waterline (WL) as shown in Fig. 1. For both cut patterns, material is removed from the workpiece in multiple passes. In the SBS cut pattern (Fig. 1a) all passes are completed for one side of the workpiece before proceeding to the other side. In the WL cut pattern (Fig. 1b), the passes for one height level are completed for both sides before proceeding to the next height level. The same axial depth of cut $a_p$ is used for all passes, except for the lowest pass that is adjusted so that 1 mm height from the fixture is left uncut. The toolpath are generated by Siemens NX version 2206.

### Cutting parameter ranges

The workpieces are machined using different cut patterns, machining allowance, cutting tools and cutting parameters, i.e. depth of cut, radial width of cut and feed rate, in order to investigate how these changes will affect the SLE on the workpiece.

A governing assumption for the method presented in this paper is that the quasi-static part of the in-process deflections are substantially larger than the dynamic counterpart. Therefore, the spindle speeds are selected such that the resulting tooth passing frequencies are much lower than the natural frequencies of the vibration modes, therefore the deformation response of the structure is mainly controlled by the stiffness of the structure, i.e. inertial forces that excite vibrations are negligible, resulting in negligible vibrations that are damped fast. Also, the measured force signals also suggest that chatter vibrations are not encountered in experiments.

In Table 2 the cutting parameter ranges, cut patterns and cutting tools are specified. Tool 1 and Tool 2 are decribed in “Model calibration”.

**Table 2:** Cutting parameter ranges.

Parameter	Range
Cutting tool	Tool 1; Tool 2
Cut pattern	SBS; WL
Radial width of cut, $a_e$ (mm)	0.2; 0.6; 1.0; 1.4
Axial depth of cut, $a_p$ (mm)	8.5; 17.0
Feed per flute, $f_z$ (mm/flute)	0.05; 0.10; 0.15; 0.20
Feed rate, $v_f$ (mm/min)	80; 160; 240; 320

## Modelling framework

**Figure 2:**
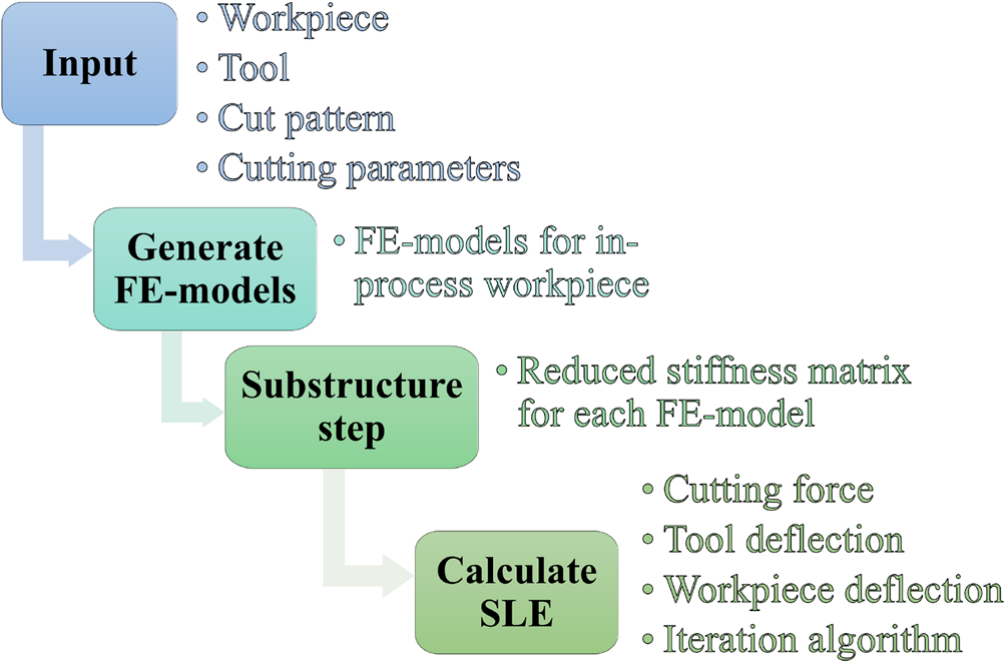
Overview of the modelling framework.

The modelling framework, depicted in Fig. 2, is used to predict the SLE on both sides of the plates and the corresponding thickness error $\Delta t$. The framework consist of four steps which are denoted Input-step, Generate FE-models-step, Substructure-step and Calculate SLE-step. In the Input-step all input parameters are assigned, which includes the workpiece geometry, cutting tool, cut pattern and cutting parameters. The remaining three steps of the framework are described in the following “Generation of FE-models”–“Prediction of the surface location error”.

### Generation of FE-models

The thin-walled workpiece is modelled using the commercial FE software Abaqus CAE 2017. During the cutting process, the stiffness of the workpiece is continously reduced due to removal of material and therefore the FE-mesh is continously modified. For each tool position, a unique FE-model is created to account for the material that has been removed prior to this position. The generation of FE-models is automated using MATLAB and Python scripts. In this work, the cutting process of each level is divided into 21 tool positions along the feed direction. The number of levels depends on the axial cutting depth and cut pattern.

Since the methodology is based on a global modelling approach, the purpose of the FE model is to model the force–displacement response of the workpiece. Therefore, the element size was selected by ensuring a converged force–displacement response along the tool/workpiece contact zone at all tool positions. The workpiece is modelled using 15-node quadratic triangular prism elements (C3D15) with an approximate element size of 1.4 mm. The workpiece material is modelled as linear-elastic with a Young’s modulus and Poisson’s ratio of 70 GPa and 0.27, respectively.

### Substructure step

**Figure 3:**
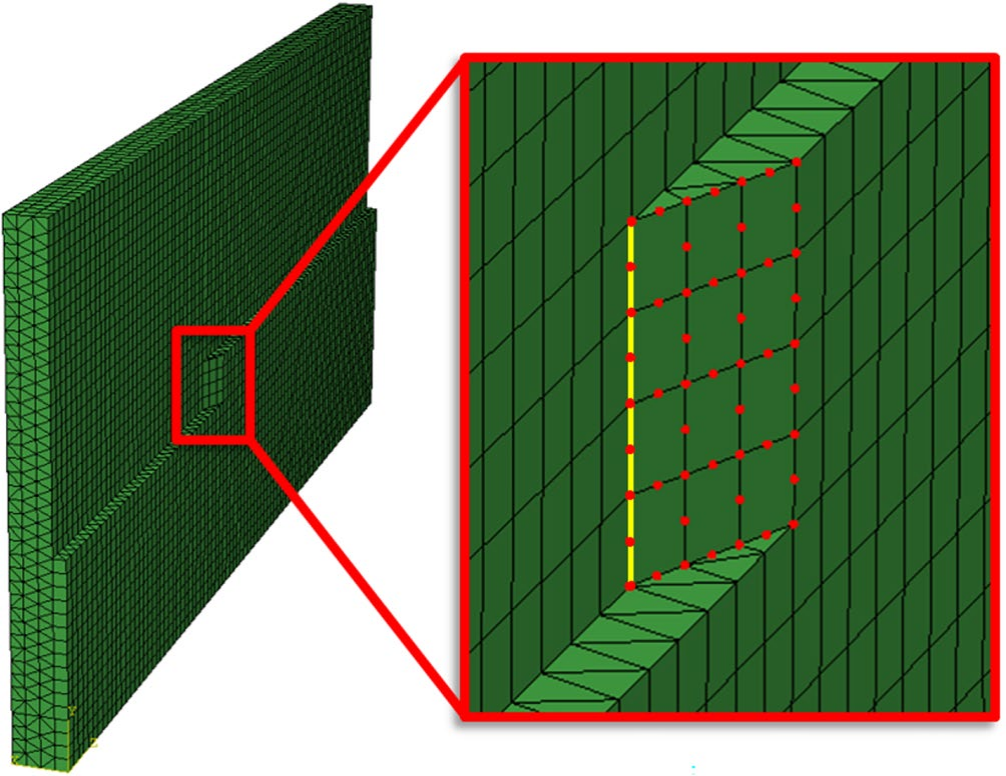
FE-model of the workpiece for a specific tool position. Red nodes: nodes that are retained. Nodes on the yellow line: surface generation points.

The radial stiffness of the workpiece at each tool position is computed in the form of a reduced stiffness matrix using a substructure step in Abaqus. Figure 3 shows the FE-model of the workpiece for a specific tool position where the reduced stiffness matrix is calculated for the highlighted nodes. Nodes on the yellow line are in the following denoted surface generation points (SGP:s) and at each tool position, finished surface is generated along this line by rotating the tool such that points on the helical flutes intersect with the nodes along the yellow line, see^39^ for a detailed description. The stiffness at the surface generation points is not significantly influenced by the geometric simplification to a flat contact surface as the displacements are governed by the applied load and global stiffness of the plate. The highlighted nodes represents potential tool-workpiece contact points at this tool position.

From the global stiffness matrix $\varvec{K}^{tp}$, corresponding to an instantaneous workpiece geometry at tool position *tp*, the reduced stiffnes matrix $\varvec{K}_{red}^{tp}$ is calculated. Since only the radial stiffness at potential tool-workpiece contact nodes are retained, the dimension of $\varvec{K}_{red}^{tp}$ is significantly smaller than the dimension of $\varvec{K}^{tp}$. The computation of the reduced stiffness matrix is included in the Python script that generates the FE-models and is in that way an automated process using the Abaqus Scripting Interface.

### Prediction of the surface location error

The SLE is calculated at each SGP through an iteration algorithm due to the interaction between the in-process radial deflections and the radial cutting forces. To predict the SLE, a reliable cutting force model, as well as structural models of the tool and instantaneous workpiece at each tool position are needed.

#### Cutting force model

**Figure 4:**
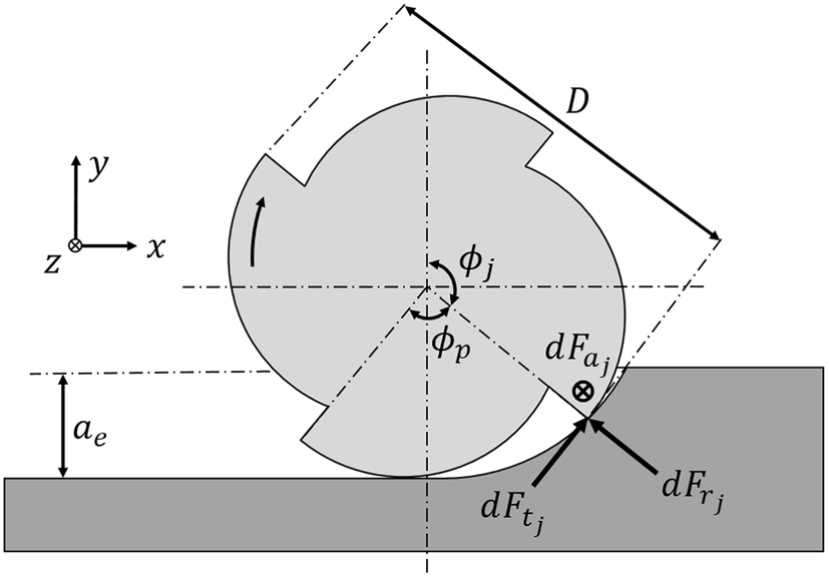
Differential cutting forces on the j:th flute of an end mill.

The mechanistic cutting force model in^40^ is used to model the cutting force and it is presented here for clarity. In this mechanistic linear-edge force model the differential cutting forces, acting on the j:th flute, in the tangential (*t*), radial (*r*) and axial (*a*) directions, depicted in Fig. 4, are given by

1 $$\begin{aligned} \begin{aligned}{}&d F_{\text {t},j} (\phi ,z) = \left[ K_{\mathrm{{te}}} + K_{\mathrm{{tc}}} h_j \left( \phi _j,z \right) \right] dz\\&d F_{\text {r},j} (\phi ,z) = \left[ K_{\mathrm{{re}}} + K_{\mathrm{{rc}}} h_j \left( \phi _j,z \right) \right] dz\\&d F_{\text {a},j} (\phi ,z) = \left[ K_{\mathrm{{ae}}} + K_{\mathrm{{ac}}} h_j \left( \phi _j,z \right) \right] dz \end{aligned} \end{aligned}$$

where $h_j \left( \phi _j,z \right)$ is the chip thickness as a function of the immersion angle $\phi _j$ for flute *j* and the axial coordinate *z*. The subscripts e and c of, $K_{\cdot \mathrm e}$ and $K_{\cdot \mathrm c}$, denote edge force coefficients and cutting force coefficients, respectively. In Fig. 4, *D*, $\phi _{\mathrm{{p}}}$ and $a_{\textrm{e}}$ are the tool diameter, tool pitch angle and the radial width of cut, respectively. The chip thickness varies with the instantaneous immersion angle and can be expressed as

2 $$\begin{aligned} h_j (\phi _j,z) = f_z \sin \phi _j (z) \end{aligned}$$

where $f_z$ is the feed per flute and $\phi _j (z)$ is given by

3 $$\begin{aligned} \phi _j (z) = \phi + (j-1)\phi _{\textrm{p}} - \frac{2 \textrm{tan} \beta }{D}z \end{aligned}$$

where $\phi$ is the reference angle, i.e. $\phi _1(0)$. The last term in Eq. (3) is the lag angle and represents the angle for which a point on the cutting edge, at axial coordinate *z*, has lagged behind the end point of the tool. This lag angle, is due to the helix angle $\beta$ of the tool.

The differential cutting forces are transformed into the *x* (feed), *y* (normal) and *z* (axial) directions using the transformation

4 $$\begin{aligned} \varvec{dF}_{xyz,j} (\phi _j (z)) = \varvec{T} \varvec{dF}_{tra,j} (\phi _j (z)) \end{aligned}$$

where the transformation matrix, $\varvec{T}$, is given by

5 $$\begin{aligned} \varvec{T} = \begin{pmatrix} -{\textrm{cos}} (\phi _j (z)) &{} -\textrm{sin} (\phi _j (z)) &{} 0 \\ {\textrm{sin}} (\phi _j (z)) &{} -\textrm{cos} (\phi _j (z)) &{} 0 \\ 0 &{} 0 &{} 1 \end{pmatrix} \end{aligned}$$

The total cutting forces are computed by integrating the differential cutting forces over the immersed part of flute *j*

6 $$\begin{aligned} \varvec{F}_{xyz,j} (\phi ) = \int _{z_{j,1}}^{z_{j,2}} \varvec{dF}_{xyz,j} (\phi _j (z)) \, dz \end{aligned}$$

where $z_{j,2}$ and $z_{j,1}$ represents the upper and lower engagement limits of the immersed part of flute *j*. As described in^39^, $z_{j,2}$ and $z_{j,1}$ are determined by the start and exit engagement angles of the cut along the tool-workpiece contact zone. In down-milling the exit engagement angle is $\phi _{\mathrm{{ex}}} =\pi$, always, and the start engagement angle is given by

7 $$\begin{aligned} \phi _{\mathrm{{st}}} = \pi - \textrm{cos}^{-1} \left( 1- \frac{2a_{\mathrm{{e}} }}{D} \right) \end{aligned}$$

The cutter is discretized into *n* cutter elements and the integrations in Eq. (6) is carried out element wise, in order to compute the elemental cutting force acting on flute *j*, $\varvec{F}_{xyz,j}^{el} (\phi )$, according to

8 $$\begin{aligned} \varvec{F}_{xyz,j}^{el} (\phi ) = \int _{\zeta _{j,1}}^{\zeta _{j,2}} \varvec{dF}_{xyz,j} (\phi _j (z)) \, dz \end{aligned}$$

Here, $\zeta _{j,2}$ and $\zeta _{j,1}$ are the local upper and lower integration boundaries for the current cutter element. These local integration boundaries are determined by first computing the global integration boundaries, $z_{j,2}$ and $z_{j,1}$, for the current tool position and immersion angle of the tool, and then comparing these values with the *z*-coordinates for the element

9 $$\begin{aligned}{}&\begin{aligned}{}&\zeta _{j,1} = z_{min}^{el} \quad (z_{j,1} \le z_{min}^{el})\\&\zeta _{j,1} = z_{j,1} \quad (z_{min}^{el} \le z_{j,1} \le z_{max}^{el}) \end{aligned} \end{aligned}$$

10 $$\begin{aligned}{}&\quad \begin{aligned}{}&\zeta _{j,2} = z_{max}^{el} \quad (z_{max}^{el} \le z_{j,2}) \\&\zeta _{j,2} = z_{j,2} \quad (z_{min}^{el} \le z_{j,2} \le z_{max}^{el}) \end{aligned} \end{aligned}$$

where $z_{min}^{el}$ and $z_{max}^{el}$ are the minimum and maximum *z*-coordinate for the current element, respectively.

The total elemental cutting force is computed by summing the force contributions from all flutes as

11 $$\begin{aligned} \varvec{F}_{xyz}^{el} (\phi ) = \sum _{j=1}^{n_f} \varvec{F}_{xyz,j}^{el} (\phi ) \end{aligned}$$

where $n_f$ is the number of flutes for the tool. The total cutting force acting on the tool is finally computed by summing the elemental cutting force for each element along the tool

12 $$\begin{aligned} \varvec{F}_{xyz} (\phi ) = \sum _{el=1}^{n} \varvec{F}_{xyz}^{el} (\phi ). \end{aligned}$$

#### Tool deflections

**Figure 5:**
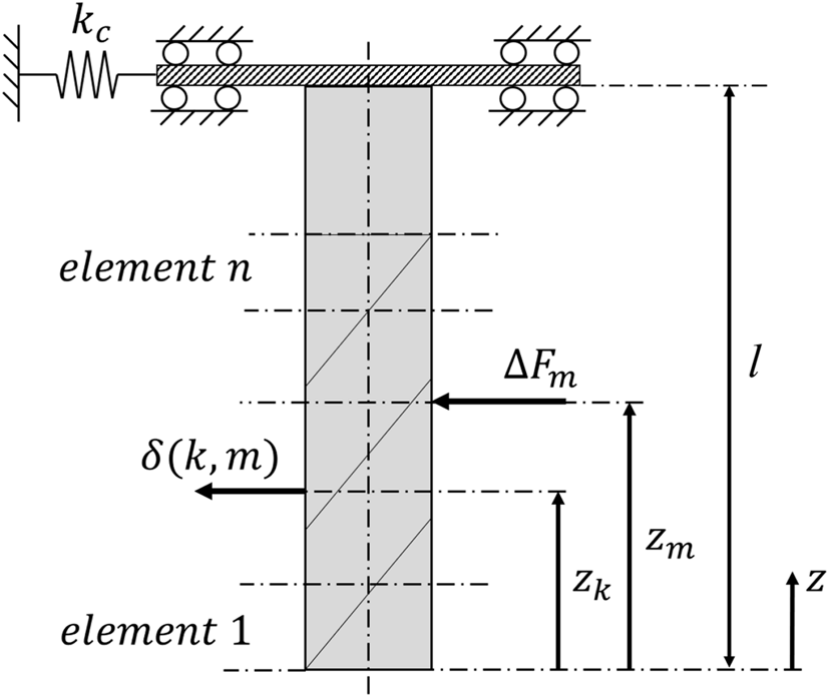
Beam model of the tool and its discretization.

As in^21^, beam theory is used to model deflections along the tool. The load case is idealized as a cantilever beam with an elastic support in the normal direction of the workpiece, see Fig. 5. The elemental cutting force are equally distributed to the respective end nodes of the cutter element. That is, the elemental radial force of element *m* is distributed equally on nodes *m* and $(m+1)$. The tool deflection at node *k* due to the radial force acting at node *m*, $\Delta F_{\mathrm{{m}}}$, is given by^39^

13 $$\begin{aligned}{} & {} \delta (k, m) = \nonumber \\{} & {} {\left\{ \begin{array}{ll} \frac{\Delta F_{\mathrm{{m}}} (l-z_{\mathrm{{m}}})^2}{6EI} (2l-3z_{\mathrm{{k}}}+z_{\mathrm{{m}}}) +\frac{\Delta F_{\mathrm{{m}}}}{k_c}, \ 0<z_{\mathrm{{k}}}<z_{\mathrm{{m}}}\\ \frac{\Delta F_{\mathrm{{m}}} (l-z_{\mathrm{{k}}})^2}{6EI} (2l-3z_{\mathrm{{m}}}+z_{\mathrm{{k}}}) +\frac{\Delta F_{\mathrm{{m}}}}{k_c}, \ z_{\mathrm{{m}}}<z_{\mathrm{{k}}}<l\\ \end{array}\right. } \end{aligned}$$

where $k_c$ is the clamping stiffness for the tool-machine interface, *l* is the gauge length for the tool, $z_{\mathrm{{m}}}$ and $z_{\mathrm{{k}}}$ are the axial coordinates of nodes *m* and *k*, respectively, *E* is the Youngs modulus for the tool material and *I* is the area moment of inertia of a circular cross-section with diameter $D_e = sD$. Here, *s* is a scaling factor that represent the reduced diameter of the tool due to the helical flutes^39^. The radial deflection at node *k* due to all nodal forces is then calculated by superposition, i.e.

14 $$\begin{aligned} \delta (k) = \sum _{m=1}^{n+1}\delta (k, m) \end{aligned}$$

#### Workpiece deflections

At each tool position, the workpiece deflection is calculated for a number of immersion angles as

15 $$\begin{aligned} \varvec{w}^{tp}(\phi ) = \left( \varvec{K}_{red}^{tp}\right) ^{-1} \varvec{F}_{wp,y}^{tp}(\phi ) \end{aligned}$$

where $\left( \varvec{K}_{red}^{tp}\right) ^{-1}$ is the inverse of the reduced stiffness matrix at tool position *tp*. The vectors $\varvec{F}_{wp,y}^{tp}(\phi )$ and $\varvec{w}^{tp}(\phi )$ denote the radial force acting on the workpiece and the normal workpiece deflection at tool position *tp*. The radial force vector consists of the cutter elemental normal forces acting in the opposite direction and they are distributed on the workpiece nodes by using weight functions.

#### Iteration algorithm for cutting force-induced form errors

The interaction of the cutting forces and the in-process deflections necessitates an iterative calculation method for the SLE^39^. Substituting $a_e$ with $a_e^f$ in Eq. (7) gives the effective start angle due to tool and workpiece deflections

16 $$\begin{aligned} \phi _{\mathrm{{st}}}^{f} = \pi - \textrm{cos}^{-1} \left( 1- \frac{2a_{\textrm{e}}^{f}}{D} \right) \end{aligned}$$

where the effective radial width of cut due to tool and workpiece deflections is given by

17 $$\begin{aligned} a_{\textrm{e}}^f(z,\phi ) = a_e+\delta (z,\phi )-w(z,\phi ) \end{aligned}$$

where $w(z,\phi )$ denotes the workpiece deflections along the yellow line in Fig. 3. That is, the in-process deflections determine the effective start angle in Eq. (16) and thereby also the upper integration boundary in Eq. (6), which in turn affects the cutting forces and the in-process deflections. Therefore, the iterative solution strategy is required to compute the forces and deflections at the equilibrium state.

The SLE at each SGP node, *k*, is finally calculated as

18 $$\begin{aligned} {\textrm{SLE}}(k) = \delta (k)-w(k) \end{aligned}$$

The SLE at all lateral surface nodes are mapped as ${SLE}^{side}(x,z)$, and the thickness error map is finally calculated as

19 $$\begin{aligned} \Delta {t}(x,z) = {SLE}^{\textrm{1}}(x,z)+{SLE}^{\textrm{2}}(x,z) \end{aligned}$$

## Model calibration

### Cutting coefficients

The cutting force coefficients in Eq. (1) are calibrated by fitting average force expressions to experimentally measured average forces through linear regression, following^21^. The calibration experiments consist of flank milling tests for workpieces made of aluminum EN AC-46000 (AlSi9Cu3(Fe)) using feed per flutes of $f_{\textrm{z}}$ = [0.050; 0.0875; 0.125; 0.1625; 0.200] mm/flute, which corresponds to feed rates of $v_{\textrm{f}}$ = [80; 140; 200; 260; 320] mm/min, axial depth of cut $a_{\textrm{p}}$ = 17 mm, radial width of cut $a_{\textrm{e}}$ = 1.4 mm and spindle speed *n* = 800 rpm. The considered tools are carbide end mills of the type Sandvik CoroMill. The tool parameters for Tool 1 (Product code: 2S221-1200-150-NG H10F) and Tool 2 (Product code: 2S221-2000-250-NG H10F) are shown in Table 3 and the corresponding cutting- and edge force coefficients in the tangential, radial and axial directions are calibrated for the considered tools to the values according to Table 4.

**Table 3:** Tool parameters.

Tool	*D* (mm)	*R* (mm)	$\beta$ (${\mathrm{^o}}$)	No. flutes
Tool 1	12	1.5	30	2
Tool 2	20	2.5	30	2

**Table 4:** Cutting force coefficients.

Tool	$K_{tc} \, \mathrm{(N/mm^2)}$	$K_{rc} \, \mathrm{(N/mm^2)}$	$K_{ac} \, \mathrm{(N/mm^2)}$	$K_{te} \, \mathrm{(N/mm)}$	$K_{re} \, \mathrm{(N/mm)}$	$K_{ae} \, \mathrm{(N/mm)}$
Tool 1	805	364	162	4.98	4.27	1.64
Tool 2	815	379	138	3.86	3.20	1.11

### Tool clamping stiffness

The parameters $k_{\mathrm{{c}}}$ and *s* in the tool deflection model described in “Tool deflections” are experimentally measured and calibrated. With the tool mounted in the machine, these measurements are performed by pressing the tool against the force transducer at various points along the tool and for various prescribed tool deflections realized by means of a machine displacement of the tool. A Young’s modulus of *E* = 620 GPa is assumed for the tool material and the gauge lengths are measured to $l_{\textrm{1}}$ = 63 mm and $l_{\textrm{2}}$ =101 mm, where the subscripted indices indicate Tool 1 and Tool 2, respectively. From the measured force-deflection relations, the identified parameters are $k_c$ = 8.36 kN/mm, $s_{\textrm{1}}$ = 0.7 and $s_{\textrm{2}}$ = 0.67.

## Experimental series

Three cutting experiments are performed to verify the predicted cutting forces and thickness errors. Table 5 shows the considered cutting parameters, cut patterns and tools for these experiments.

**Table 5:** Verfication experiments 1–3.

Exp	Tool	Cut pattern	$a_e$ $\mathrm{(mm)}$	$a_p$ $\mathrm{(mm)}$	$f_z$ $\mathrm{(mm/flute)}$	$v_f$ $\mathrm{(mm/min)}$
1	Tool 1	SBS	1.4	17	0.2	320
2	Tool 1	WL	1.0	17	0.05	80
3	Tool 2	SBS	0.6	17	0.05	80

### Materials and workpiece preparation

The workpieces are manufactured from aluminum EN AC-46000 (AlSi9Cu3(Fe)) ingots using water jet cutting. Before each experiment, the workpiece is also pre-machined with conservative cutting parameters in order to align the workpiece with the machine axes. The initial thickness of the workpiece is adapted to the considered radial width of cut, as the final thickness should be 3 mm, i.e. the initial thickness is 2$a_e$+3 mm. Before each experiment the initial thickness of the workpiece is carefully measured at multiple locations using a spindle mounted probe and an initial thickness error of less than 50 $\upmu \text {m}$ is recorded for all workpieces.

### Experimental setup

**Figure 6:**
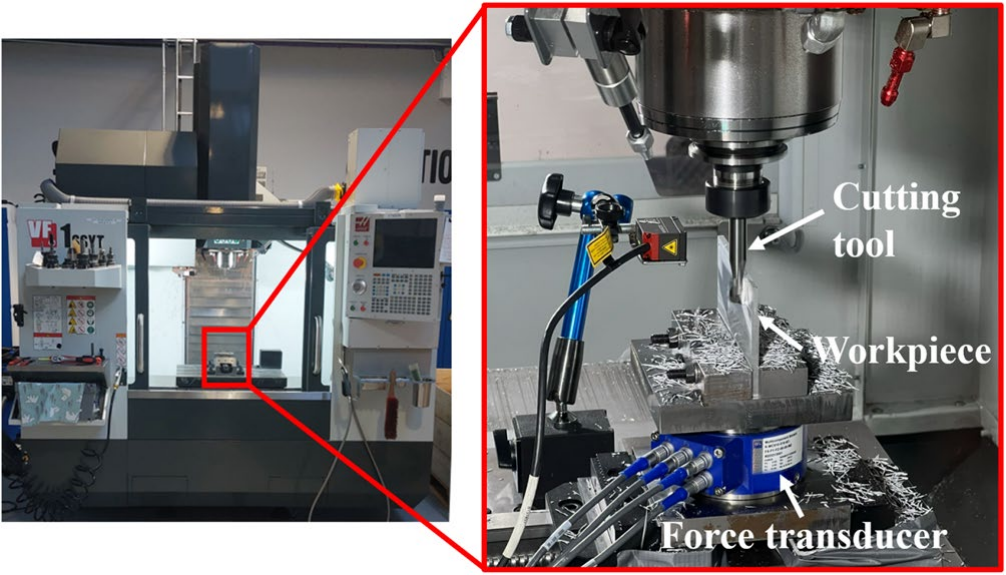
Experimental setup including Haas VF-3SS machining center, cutting tool, force transducer and a mounted workpiece.

The cutting forces are measured using an HBM MCS10 multicomponent force transducer. The force signals are amplified and acquired using HBM ClipX measuring amplifiers, a National Instruments USB 6210 data acquisition card together with the data acquisition tool box in MATLAB. Both pre-machining of workpieces and actual experiments are conducted in a vertical machining center of the type Haas VF-3SS. The thickness errors, are measured using a coordinate measuring machine of the type Hexagon DEA Global Advantage 15.20.10 with a resolution of 0.058 $\upmu \text {m}$. Figure 6 shows the experimental setup, where the workpiece is mounted in the Haas VF-3SS machining center, together with a cutting tool and the force transducer.

## Comparison of experimental results with simulations

The predicted cutting forces and thickness errors are compared with the experimentally measured counterparts.

**Figure 7:**
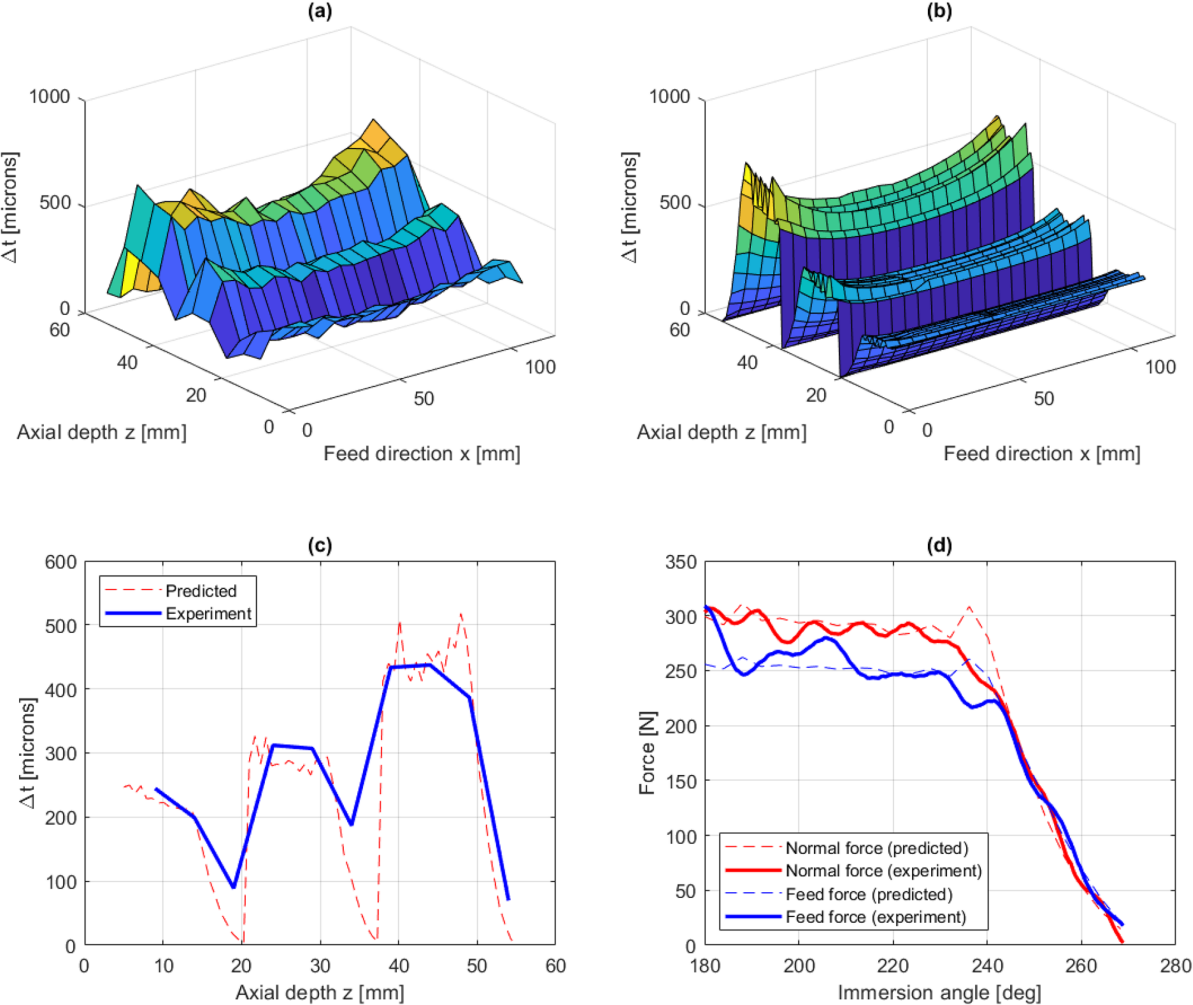
Experiment 1: measured thickness errors (**a**), predicted thickness errors (**b**), predicted and experimentally measured thickness errors along $x =$ 54 mm (**c**) and comparison between predicted and measured cutting forces (**d**).

In Fig. 7, predicted and experimentally measured results are shown for experiment 1. The experimental and predicted thickness errors are shown in Fig. 7a,b. Here, and in the following thickness error plots, the z-coordinate corresponds to the axial direction of the plate, where $z =$ 0 and $z =$ 55 mm corresponds to the fixed and free end of plate, respectively. The maximum thickness error is predicted to occur near the top of the workpiece, where the tool entries the material in the first pass, due to the low workpiece stiffness correlated to this tool position. The thickness error at this tool position is not experimentally measured due to difficulties in measuring the thickness at the corner of the workpiece. Instead, the first measuring point is placed 1 mm from the corner. In the axial direction, the first measuring point is placed 10 mm from the fixed end, due to irregularities that may arise in the interface between machined and unmachined surfaces.

From the experimental data, the maximum thickness error occurs at a tool position of $x =$ 6.9 mm in the feed direction, with a value of 670 $\upmu$m. Comparing this with the predicted thickness error of 683 $\upmu$m at the same location, gives a prediction error of less than 2%. From Fig. 7a,b it can be seen that the proposed modelling framework successfully predicts the overall distribution of the thickness error. Furthermore, Fig. 7c shows a comparison between the predicted and experimentally measured thickness error along a vertical line at $x =$ 54 mm, i.e. in the middle of the workpiece. A close agreement is found between the predicted and measured thickness error. In Fig. 7d, the cutting force is plotted against the immersion angle at the instant when the tool is positioned at the top level approximately 6 mm in the feed direction. Again, there is a close agreement between the predicted and measured forces.

**Figure 8:**
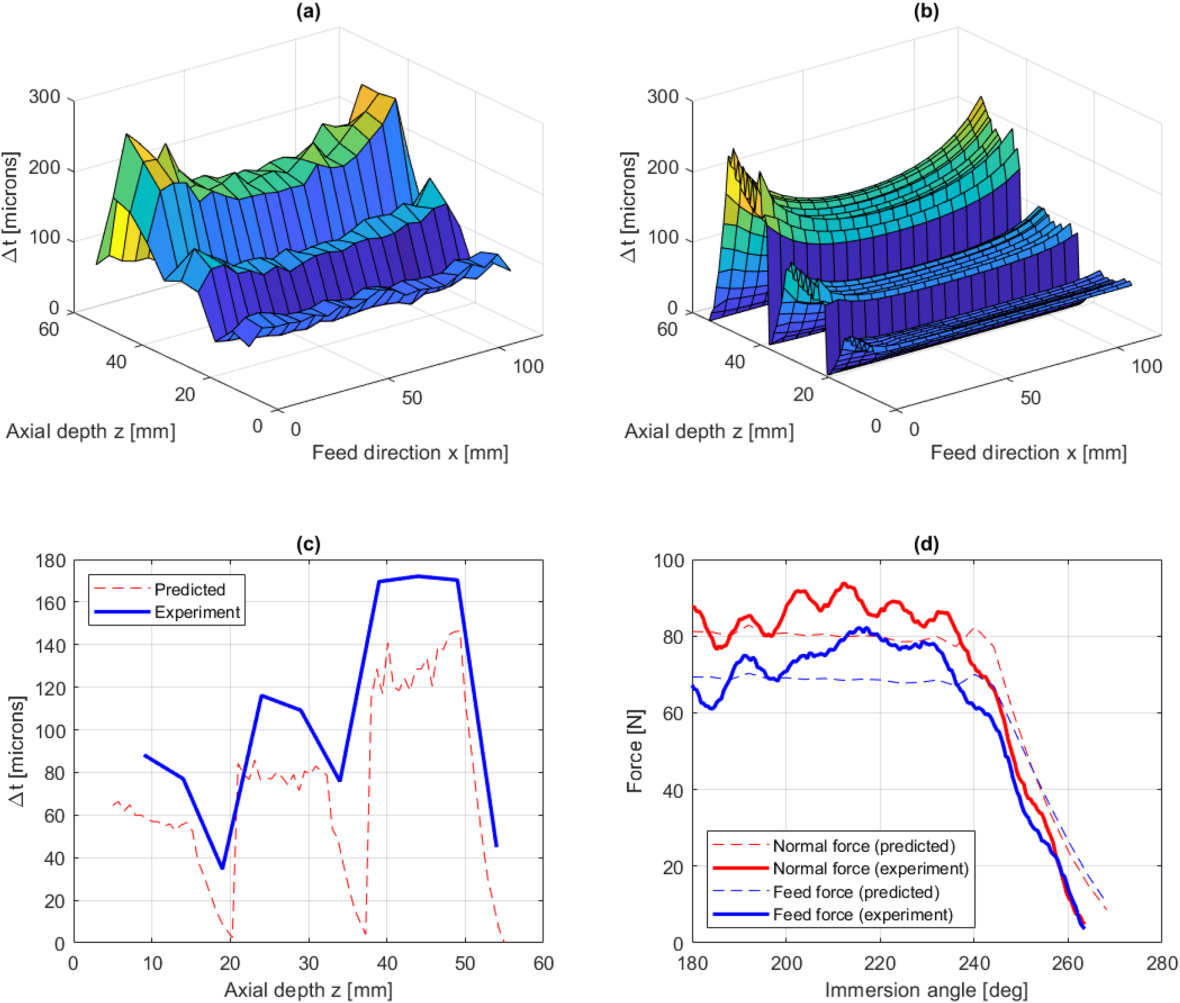
Experiment 2: measured thickness errors (**a**), predicted thickness errors (**b**), predicted and experimentally measured thickness errors along $x =$ 54 mm (**c**) and comparison between predicted and measured cutting forces (**d**).

In Fig. 8a,b, predicted and experimentally measured thickness errors are shown for experiment 2. Similar to experiment 1, where the SBS cut pattern was adopted, the maximum thickness error occurs at the top of the workpiece where the tool entries the material in the first pass. From the experimental data, the maximum thickness error is measured to 273 $\upmu$m. This can be compared with the predicted thickness error at the same tool position, with a maximum value of 261 $\upmu$m, which gives a prediction error of about 4%. Furthermore, Fig. 8c shows a comparison between predicted and experimentally measured thickness errors along the line $x =$ 54 mm. Here, the overall distribution is well captured but the predicted thickness error is underestimated with about 20–40%. A close agreement between predicted forces and experimentally measured forces is obtained, as shown in Fig. 8d.

**Figure 9:**
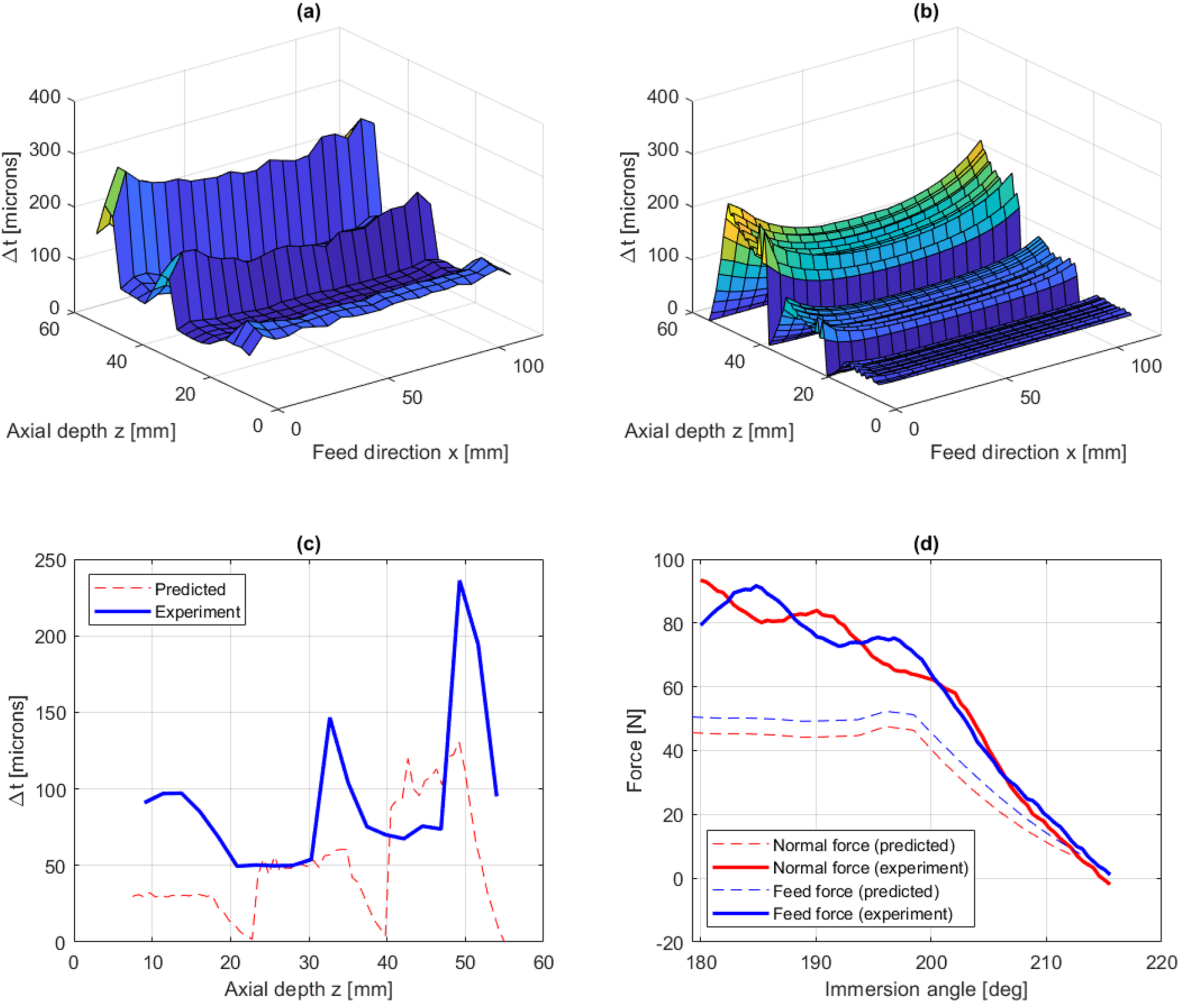
Experiment 3: measured thickness errors (**a**), predicted thickness errors (**b**), predicted and experimentally measured thickness errors along $x =$ 54 mm (**c**) and comparison between predicted and measured cutting forces (**d**).

For experiment 3, measured and predicted thickness errors are shown in Fig. 9a,b. The maximum thickness error occurs at the top of the workpiece, where the tool enters the material in the first pass, similar to the cases with Tool 1. The measured maximum thickness error is about 293 $\upmu$m. This can be compared with the predicted thickness error at the same position of 222 $\upmu$m, which gives a prediction error of about 24%.

Figure 9c, shows a comparison between predicted and experimentally measured thickness errors along $x =$ 54 mm. As shown, the cutting depth in level 1 is smaller than 17 mm due to the large nose radius/radial depth of cut ratio. Large discrepancies between measured and predicted values are observed. The thickness error in experiment 3 was measured using more measuring points along the z-direction, specifically 20 points instead of the previous 10. This discrepancy is believed to be explained by a re-cutting process that is not modelled in the simulations. For example, during cutting of level 2 with $a_p$=17 mm, the fluted part of the tool ranges between the lowest point of level 2 to 9 mm above the top of level 2. Since a thickness error was generated during the cutting of level 1, re-cutting takes place within level 1. Therefore, the measured thickness error in the re-cutting region of height 9 mm shows a valley that is not predicted by the model.

## Numerical evaluation of cutting strategies

After the experimental verification, the proposed modelling framework is used to investigate the effect of the free parameters, i.e. the cut pattern, machining allowance, cutting tool and cutting parameters, on the resulting thickness error. Six simulations are conducted where the free parameters are varied according to table 6. For comparison, the average value and standard deviation are calculated for the complete thickness error map for each simulation. The thickness errors are arranged in a matrix $\Delta t_{ij}$ where the rows (*i*) and the columns (*j*) corresponds to the location in *x*- and *z*-direction, respectively. The average value of the thickness errors is calculated as

20 $$\begin{aligned} \overline{\Delta t} = \frac{1}{N_x N_z}\sum _{i=1}^{N_x} \sum _{j=1}^{N_z} \Delta t_{ij} \end{aligned}$$

where $N_x$ and $N_z$ are the number of locations in the *x*- and *y*-direction, respectively. The standard deviation is calculated as

21 $$\begin{aligned} \sigma = \sqrt{\frac{1}{N_x N_z}\sum _{i=1}^{N_x} \sum _{j=1}^{N_z} (\Delta t_{ij}-\overline{\Delta t})^2} \end{aligned}$$

**Table 6:** Input parameters end results for simulation 1–6.

Sim	Tool	Cut pattern	$a_e$ $\mathrm{(mm)}$	$a_p$ $\mathrm{(mm)}$	$f_z$ $\mathrm{(mm/flute)}$	$v_f$ $\mathrm{(mm/min)}$	max$(\Delta t)$ ($\upmu$m)	$\overline{\Delta t}$ ($\upmu$m)	$\sigma$ ($\upmu$m)
1	1	SBS	1.4	8.5	0.05	80	357	74	67
2	1	WL	1.4	8.5	0.05	80	264	65	54
3	2	WL	1.4	8.5	0.05	80	207	37	34
4	2	WL	0.6	8.5	0.05	80	247	46	42
5	2	WL	0.6	17	0.05	80	211	58	42
6	2	WL	0.6	17	0.2	320	428	134	92

## Results

**Figure 10:**
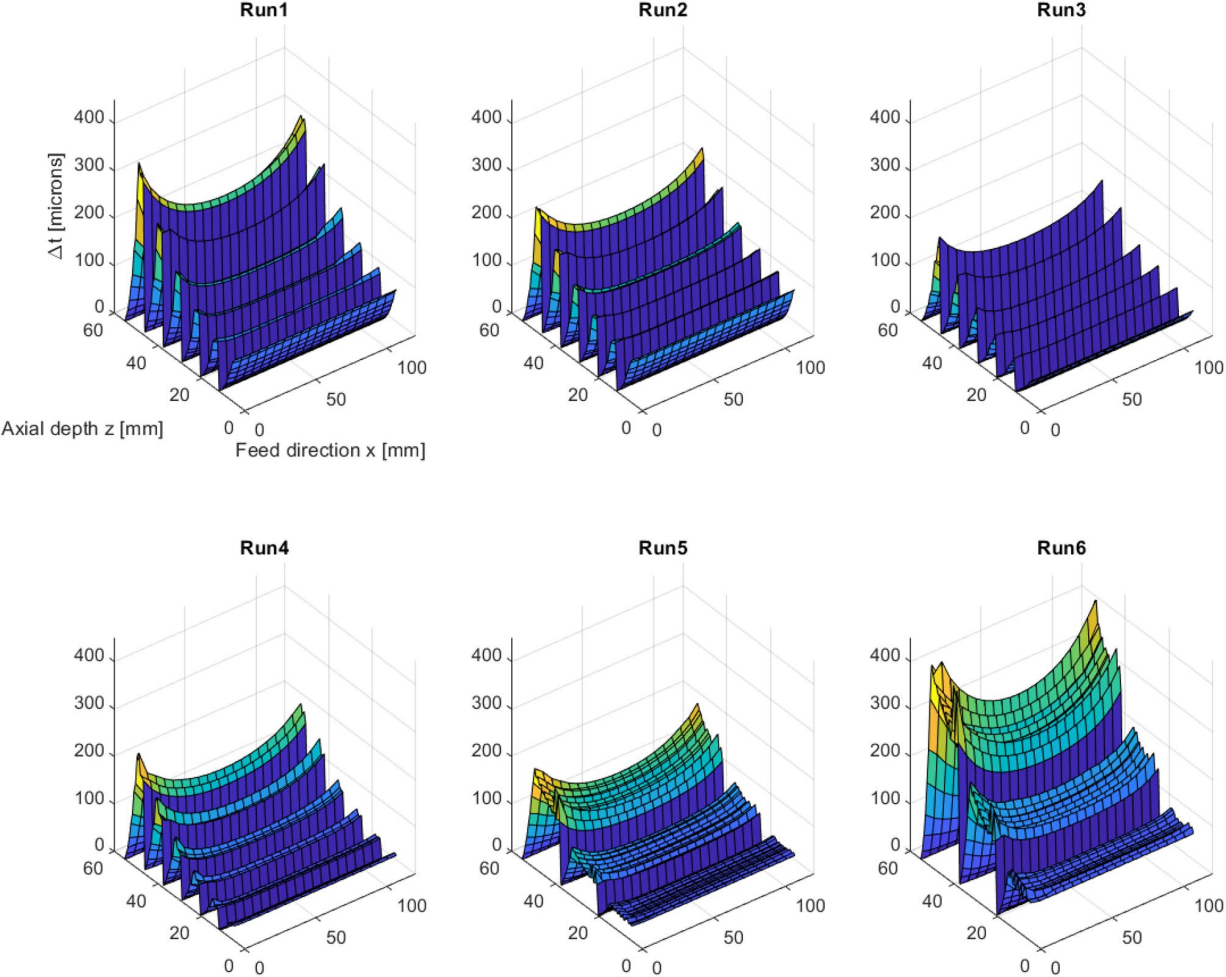
Predicted thickness errors in simulations 1–6.

In Fig. 10, the predicted thickness errors are shown and Table 6 shows the corresponding standard deviation and average values of the thickness errors. For comparison, Table 6 also shows the maximum thickness error for each simulation. From these results the effect of cut pattern, machining allowance, cutting tools and cutting parameters on the resulting thickness errors can be examined. Simulation 1 and 2 have the same cutting parameters except for the cut pattern. A significantly better machining accuracy (both in maximum value, average value and standard deviation) is predicted for the WL cut pattern. Comparing simulation 2 with simulation 3, it can be seen that Tool 2 (with a tool diameter of 20 mm) gives somewhat smaller thickness errors compared to Tool 1 (with a tool diameter of 12 mm). Furthermore, simulations 3 and 4 shares the same cut pattern and cutting parameters except for the radial width of cut. These results show that when the radial width of cut (or the machining allowance) is increased from 0.6 mm to 1.4 mm the maximum thickness error is decreased from 247 $\upmu$m to 207 $\upmu$m. The effect of the axial depth of cut on the thickness error can be examined by comparing simulation 4 and 5. This shows that when the axial depth of cut is increased from 8.5 mm to 17 mm, the thickness error is decreased in maximum value, but increased and unchanged in average value and standard deviation. Finally, comparing simulation 5 and 6 shows that a larger feed rate gives larger thickness errors, as expected.

## Discussion

From the simulation results presented in section 8, some key findings can be stated; (1) the WL cut pattern gives smaller thickness errors compared to the SBS cut pattern; (2) Tool 2, with a tool diameter of 20 mm, gives smaller thickness errors compared to Tool 1, which has a tool diameter of 12 mm; (3) larger machining allowances, or radial widths of cut, give smaller thickness errors ; (4) a larger axial depth of cut decreases the maximum value of the thickness error but results in a larger average value of the thickness errors over the whole workpiece and (5) an increased feedrate gives increased thickness errors.

Finding 1 can be explained by the different material removing sequences for the SBS and WL cut patterns. Compared with the SBS cut pattern, where all material is removed from one side before proceeding to the other side, the WL cut pattern preserves the stiffness of the workpiece in a better way when material is removed by alternating between sides of the workpiece. However, this effect of a preserved stiffness is much more tangible for larger machining allowances, or radial width of cuts.

Regarding finding 2, this can simply be explained by the difference in bending stiffness for the tools. Due to the larger tool diameter for Tool 2, the bending stiffnes is increased and in that way the tool deflection is decreased.

Since the final thickness of the workpieces, i.e. the thickness after machining, is the same for all simulations, a higher machining allowance, or radial width of cut, results in a stiffer workpiece during the machining process. This explains the low thickness errors observed for high machining allowances, referred to as finding 3 above. However, numerical evaluations of the SBS cut pattern shows that the effect of decreased thickness errors for larger machining allowances is much less noticeable for the SBS cut pattern compared with WL.

Finding 4, which refers to a larger average value of the thickness error over the whole workpiece for larger axial depth of cuts, can be explained by the larger cutting force associated with a large axial depth of cut. Also, the decreased number of cutting levels will effect the average value of the thickness error due to low deviation values between levels, as seen in Fig. 9.

At last, finding 5 is explained by the larger cutting forces associated with larger feed rates. In this way both the tool and workpiece deflections is increased which results in larger thickness errors.

The reason for the underestimated process forces for experiment 3, and thereby also the underpredicted form errors, are believed to stem from the simplification that the cutting tool is cylindrical. In experiment 3, both the larger tool nose radius and the smaller radial width of cut affect the cutting process such that wider and thinner chips are produced than predicted with a cylindrical tool geometry. This is described and demonstrated in e.g.^41^. Additionally, since coated carbide inserts were used, the cutting-edge radius can be assumed to be relatively large compared to the thickness of the thinner part of the chip. Consequently, larger friction forces are generated than what this simplified cutting force model predicts.

The cylindrical assumption is employed to facilitate the efficient iteration scheme presented in this paper. Taking the nose radius of the tool into account necessitates calculation of cutting forces in multiple distinct regions of the tool and the radial width of the cut would have to be separately addressed in these regions. Consequently, in-process deflection would affect each region differently. Furthermore, the elemental forces in the x-, y-, and z-directions would each require individual computation. This segmentation of tool regions would not align with the Newton-Raphson-inspired iteration scheme we employ.

## Conclusions

In this paper, a modelling framework is proposed for prediction of cutting force induced form errors during flank milling of a thin-walled workpiece. It is shown that the proposed modelling framework is able to predict thickness errors and cutting forces for thin-walled workpieces that are cut on both sides and where material is removed in multiple passes. It is important to highlight that this framework is not restricted to the studied workpiece geometry or a specific fixturing solution. The modelling framework is used to numerically evaluate the effect of different cutting strategies on the resulting thickness errors during machining of thin-walled parts. From the numerical evaluation some conclusions are drawn:

- The Waterline cut pattern gives smaller thickness errors compared to the Side by side cut pattern.
- A larger tool diameter gives smaller thickness errors compared to a small tool diameter.
- A larger machining allowance gives smaller thickness errors.
- A larger axial depth of cut decreases the maximum value of the thickness error but results in a larger average value of the thickness errors over the whole workpiece.

Furthermore, this study shows that the proposed modelling framework is capable to use for investigations of the effect of different cutting strategies on the resulting form error during machining of thin-walled components. Even though the model is capable of quantitatively predict the form error in the complete parameter range, the results in experiment 3 suggests that the validity of the force prediction can become challenging when tools with a large nose radius are used in conjunction with a small radial width of cut. In those conditions, the model generate underpredicted normal and feed forces and, as a consequence, also underpredicted form errors.

For industrial use the proposed modelling framework can be used to optimize the machining process of thin-walled components by combining the modelling framework with an optimization scheme. More clearly, there is a potential to find optimized cutting strategies, including different tools, cutting parameters and cut pattern that minimize the thickness error. Another potential with this modelling framework is that it can be extended with a tool path compensation algorithm. With such an algorithm, an optimized tool path, with respect to a minimized thickness error, can be obtained.

## Data Availability

The data used and/or analysed during the current study are available from the corresponding author on reasonable request.
